# Characterization of Metabolites in Plant-Based Milk Yogurt Enriched with *Wolffia globosa* to Improve Bionutritional and Functional Properties

**DOI:** 10.3390/ijms27104256

**Published:** 2026-05-10

**Authors:** Sukrita Punyauppa-Path, Nonthiwat Taesuk, Sujira Maneerat, Priyapa Najomtien, Pongpat Kiatprasert, Watchara Kanchanarach, Nattawadee Kanpipit, Srisan Phupaboon

**Affiliations:** 1 Department of Mathematics and Science, Faculty of Agriculture and Technology, Rajamangala University of Technology Isan Surin Campus, Surin 32000, Thailand; phira.bo@rmuti.ac.th (S.P.-P.); pongpat.ki@rmuti.ac.th (P.K.); 2 Department of Biology, Faculty of Science, Mahasarakham University, Mahasarakham 44150, Thailand; nonthiwat.t@msu.ac.th (N.T.); sujira.m@msu.ac.th (S.M.); priyapa.n@msu.ac.th (P.N.); 3 Faculty of Public Health, Mahasarakham University, Mahasarakham 44150, Thailand; watchara.k@msu.ac.th; 4 Department of Pharmaceutical Chemistry, Faculty of Pharmaceutical Sciences, Khon Kaen University, Khon Kaen 40002, Thailand; natawadee.k@kkumail.com

**Keywords:** nutriomics, LC-QTOF analysis, in silico study, ADME profiles, superfoods, plant-based yogurt, functional foods

## Abstract

Riceberry rice milk (RBRM) is rich in phytochemicals, particularly anthocyanins, which are known for their potential in managing type 2 diabetes (T2D). This study aimed to develop a novel RBRM-based yogurt derived from its polysaccharide and protein components and to evaluate the effects of supplementation with *W. globosa* powder (WGP) at 0% (F1, control), 5% (F2), 10% (F3), and 15% (F4) on nutritional and functional properties. Among all formulations, F4 exhibited the highest nutritional values, including dietary fiber (41.25%), curd protein (21.34%), and carbohydrate (starch) content (25.25%), with a lower fat content (2.13%) compared to other groups. In terms of antioxidant activity, F4 showed high total phenolic content (33.70 mg GAE/g) and total flavonoid content (25.2 mg QUE/g), along with strong radical scavenging activities, with DPPH and ABTS inhibition values of 41.52% and 78.18%, respectively. Furthermore, F4 demonstrated notable antidiabetic potential through α-amylase and α-glucosidase inhibition, with IC_50_ values of 0.89 and 1.32 mg/mL, respectively. Widely targeted metabolomics analysis identified 88 differential metabolites between F4 (potent condition) and F1 (control group). Twelve selected compounds from RBRM–WGP yogurt contributed to increased levels of amino acids, peptide derivatives, saccharides, organic acids, polyphenols, and flavonoids. Molecular docking analysis revealed that key metabolites, including vignatic acid B, glimepiride, and indoramin, exhibited strong binding affinities with the active sites of α-amylase (PDB: 2GVY, *Aspergillus niger*) and α-glucosidase (PDB: 3A4A, *Saccharomyces cerevisiae*). These findings indicate that phytonutrient compounds, particularly indoramin, play a significant role in enhancing the nutritional composition and functional properties of RBRM–WGP yogurt for potential applications in food processing.

## 1. Introduction

Plant-based foods have rapidly evolved from niche alternatives to mainstream dietary choices, driven by strong market growth, product innovation beyond meat analogues, and diversification of food offerings Sustainability concerns, health awareness, and the increasing adoption of flexitarian lifestyles have further escalated global consumer demand [1]. In alignment with these trends, the Food and Agriculture Organization (FAO) has emphasized the importance of plant-based and functional foods in achieving the 2030 Agenda for Sustainable Development [2]. The FAO serves as the official custodian agency for 21 Sustainable Development Goal (SDG) indicators and a contributing agency for an additional five, including those related to food systems and health-promoting consumption [3]. As a result, contemporary consumers increasingly prioritize foods that offer nutritional and health benefits, particularly for the prevention of non-communicable diseases associated with poor dietary [4]. Functional foods represent a promising response to these consumer demands, as they contain bioactive compounds that confer health benefits. These compounds may be naturally present in plant-based protein sources or incorporated as functional ingredients. In Southeast Asia, and particularly in Thailand, plant-based alternatives such as plant-based milks, riceberry rice, and watermeal (*W. globosa*) have attracted growing interest as functional food ingredients due to their nutritional value and sustainability potential [5,6,7,8].

Riceberry rice (*Oryza sativa* L.) is a crossbreed of Kao Hom Nin (non-glutinous purple rice) and Khoa Dawk Mali 105 (fragrant white rice), which is characterized by its high anthocyanin content and rich nutritional profiles, including dietary fiber, vitamins, minerals, and antioxidant capacity [5]. Riceberry rice also contains gamma-aminobutyric acid (GABA), a non-protein amino acid that functions as an inhibitory neurotransmitter in the brain. Consumption of GABA has been linked to various health benefits such as stress reduction, improved sleep quality, blood pressure regulation, inhibition of cancer cell proliferation, and potential protective effects against type 2 diabetes [7]. Watermeal (*W. globosa* (Roxb.) is another emerging functional ingredient and is widely recognized as a nutrient-dense superfoods. It is rich in protein, dietary fiber, essential amino acids, and various bioactive compounds, including total phenolics, flavonoids, chlorophyll, and β-glucans [9]. Under optimum conditions, watermeal can contain more than 41% protein, along with carbohydrates such as resistant starch and dietary fibers, polyunsaturated fatty acids (notably α-linolenic acid, up to 30%), and phenolic compounds with antioxidant activities [9,10]. Traditionally, watermeal has been consumed for generations in Northeastern Thailand and neighboring regions, including the Lao People’s Democratic Republic, where it is incorporated into soups, salads, and other local dishes [11]. More recently, watermeal has been adapted into modern culinary applications such as toppings for desserts and functional food products, reflecting its versatility and consumer acceptance [10,11]. In addition, fermented foods, particularly yogurt products produced through lactic acid bacteria (LAB) fermentation, are well established as functional foods due to their rich content of proteins, polysaccharides, probiotics, and bioactive metabolites [12]. These products have been reported to exhibit a wide range of physiological benefits, including antioxidant activity, reduced cholesterol, immunomodulation, antitumor, antidiabetic properties, and antihypertensive effects [13]. The integration of plant-based substrates into yogurt formulations further expands their functional potential by introducing additional phytochemicals, prebiotics, and bioaccessible nutrients [14].

Thus, the present study investigated the development of plant-based yogurt formulated from riceberry rice milk (RBRM) supplemented with varying ratios of *W. globosa* powder (WGP). The study aimed to evaluate the nutritional composition, antioxidant capacity, and antidiabetic potential of the resulting products, with particular emphasis on bioaccessibility and functional metabolites. A widely targeted metabolomics approach using liquid chromatography–mass spectrometry (e.g., UPLC-QTOF) was employed to characterize changes in metabolite profiles. Furthermore, selected phytochemicals were subjected to in silico molecular docking analysis using the SwissDock (v2024) platform, and their absorption, distribution, metabolism, and excretion (ADME) properties were predicted using the SwissADME (v2024) server.

## 2. Results

### 2.1. Nutritional Composition and Biological Activities of RBRMY-WGP Hydrolysates

**Figure 1:**
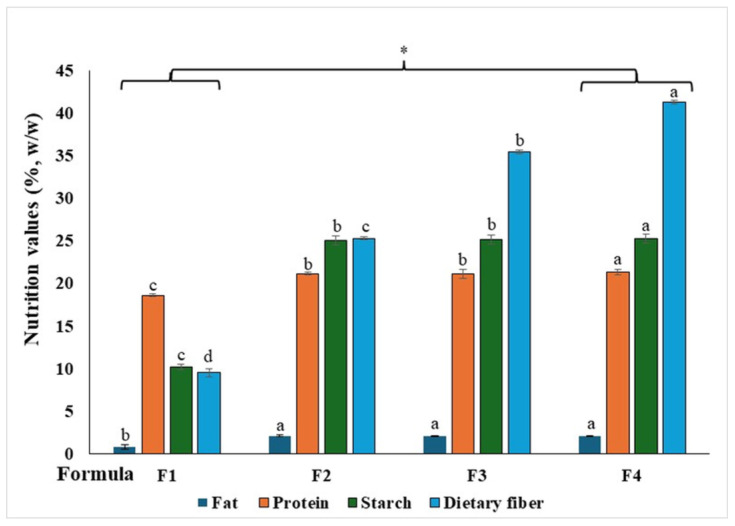
Nutritional composition of RBMY-WGP hydrolysates. Data are expressed as mean ± SD (*n* = 3). Different letters (a, b, c, d) within the same column indicate statistically significant differences (*p* < 0.05). An asterisk (*) indicates a significant different compared with the control (*p* < 0.01).

The nutritional compositions of RBRMY-WGP hydrolysates with different proportions of *W. globosa* powder (F1–F4) is presented in Figure 1. Among the formulations, the yogurt hydrolysate supplemented with 15% WGP (F4) showed significantly different nutritional characteristics (*p* < 0.05) compared with the control formulation (F1). Across all formulations, fat content ranged from 0.81 to 2.13% dry weight (DW), protein content from 18.61 to 21.34% DW, starch content from 10.20 to 25.25% DW, and dietary fiber content from 9.55 to 41.25% DW. Overall, F4 exhibited the highest protein and dietary fiber contents while maintaining the lowest fat content among all treatments. In addition, the biological activities of the RBRMY-WGP hydrolysates, including total phenolic content (TPC), total flavonoid content (TFC), antioxidant activity, and antidiabetic potential, are summarized in Table 1. The TPC values increased progressively with increasing WGP supplementation, reaching 33.70 mg GAE/g DW in F4, compared with 20.09 mg GAE/g DW in F1. A similar trend was observed for TFC, which ranged from 11.13 mg QUE/g DW in F1 to 25.20 mg QUE/g DW in F4. Antioxidant activity, evaluated using DPPH and ABTS radical scavenging assays, also increased with WGP content. DPPH inhibition ranged from 33.03% to 41.52%, while ABTS inhibition increased markedly from 12.12% in F1 to 78.18% in F4.

The antidiabetic activity of RBRMY-WGP hydrolysates was assessed via inhibition of α-amylase and α-glucosidase (Table 1). The IC_50_ values for α–amylase activity inhibition lowered from 2.42 to 0.89 mg/mL, while those for α-glucosidase inhibition decreased from 1.56 to 1.32 mg/mL across F1–F4. Although higher IC_50_ values indicate lower inhibitory potency, F4 consistently exhibited significantly improved overall biological activity compared with the other formulations, reflecting its enriched phytochemical composition. The most notable finding of this study is the limited existing evidence on the application of RBRM in yogurt fermentation supplemented with *W. globosa* as a functional protein source. The enhanced nutritional value and bioactive properties observed in F4 can be attributed, at least in part, to the synergistic effects of anthocyanins from riceberry rice and bioactive compounds from *W. globosa*.

### 2.2. UPLC-QTOF Analysis of Phytochemical Compounds in RBRMY-WGP Hydrolysates

UPLC-QTOF analysis was performed to compare the phytochemical profiles of the control formulation (F1) and the RBRMY-WGP hydrolysate containing 15% *W. globosa* powder (F4). A total of 1188 compounds were detected in negative ion mode and 989 compounds in positive ion mode (Figures S1 and S2). After excluding 88 background compounds common to both samples based on ionization mode, retention time, mass spectra, and molecular formula (Table S1), the remaining metabolites were further analyzed for their potential biological and pharmacological activities, particularly antioxidant, anticancer, and antidiabetic properties. Based on compound annotation and literature screening, twelve representative phytochemical compounds were selected from the RBRMY-WGP hydrolysates for further investigation. These included leucodelphinidin 3-O-α-L-rhamnopyranoside, macaflavone II, acarbose, vignatic acid B, cerivastatin, dihydroresveratrol, embelin, glimepiride, nateglinide, acitretin, mangostenone B, and indoramin, as identified by UPLC-QTOF and corresponding PubChem IDs.

### 2.3. Computational Results of Phytochemical Compounds in RBRMY-WGP Hydrolysates

**Figure 2:**
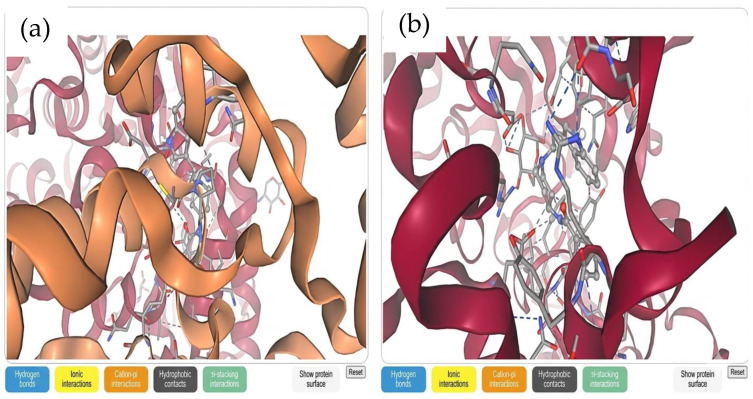
Three-dimensional molecular docking models of indoramin as a high-potential ligand-binding protein active site interaction between; (**a**) α-amylase (PDB: 2GVY, *A. niger*) and (**b**) α-glucosidase (PDB: 3A4A, *S. cerevisiae*).

To further evaluate the antidiabetic potential of the selected compounds, molecular docking and in silico analyses were performed to assess their binding interactions with α-amylase (PDB ID: 2GVY obtained from *A. niger*) and α-glucosidase (PDB ID: 3A4A obtained from *S. cerevisiae*). Docking results are summarized in Table 2 and illustrated in Figure S3. The calculated binding affinities ranged from −5.396 to −9.867 kcal/mol for α-amylase and from −1.126 to −8.350 kcal/mol for α-glucosidase. Among the evaluated compounds, indoramin (PubChem ID: 33625), glimepiride (PubChem ID: 483928029), and vignatic acid B (PubChem ID: 85261327) exhibited strong binding affinities toward both enzyme targets. Notably, indoramin demonstrated the highest predicted binding affinity, with values of −9.155 kcal/mol for α-amylase and −8.216 kcal/mol for α-glucosidase, as illustrated in Figure 2.

### 2.4. Physicochemical Properties and Pharmacokinetic Profiles

In silico prediction of physicochemical and pharmacokinetic properties was performed using the SwissADME server for three selected ligands, including indoramin, glimepiride, and vignatic acid B, identified from RBRMY-WGP hydrolysates. These compounds were evaluated in relation to their interactions with the antidiabetic target enzymes α-amylase (PDB ID: 2GVY) and α-glucosidase (PDB ID: 3A4A). The predicted molecular descriptors, including physicochemical characteristics, pharmacokinetic parameters, and drug-likeness properties, are summarized in Table 3. The results indicated that the selected compounds possessed molecular weights ranging from 347.45 to 519.63 g/mol and contained 2–7 hydrogen bond donors and/or acceptors. Lipophilicity values (consensus Log Po/w) ranged from 2.09 to 3.43, while predicted aqueous solubility (Log S) ranged from −4.46 to −4.98. All three compounds demonstrated high predicted gastrointestinal absorption, with varying degrees of blood–brain barrier (BBB) permeability, and complied with Lipinski’s rule of five, indicating favorable drug-likeness profiles. Furthermore, the molecular structures of three compounds with biological effects include indoramin, a tryptamine classified as an alpha-1 adrenergic receptor antagonist (alpha-blocker); glimepiride, an oral antidiabetic agent from the sulfonylurea class; and vignatic acid B, a naturally occurring cyclic peptide compound.

## 3. Discussion

During the past 30–40 years much more information has become available on fruit and/or plant colors (e.g., red, orange, pink, black, green, and purple), especially a purple color, as anthocyanins are well recognized for their antioxidant and antidiabetic properties, contributing to improved metabolic health [5]. Additionally, green is the most common color found in plants, particularly in *Wolffia* sp., which serves as a substitute protein source due to its exceptional nutritional composition, making it a significant focus of this research.

A comparable finding has been reported for farm-grown *Wolffia*-1, which exhibited high protein content (22.7%), dietary fiber (16.5%), TFC (5.0 mg QE/g DW), and TPC (3.9 mg GAE/g DW), along with elevated levels of total amino acids [9]. Furthermore, several studies on Thai plant-based milk alternatives, including red rice and green rice milks, have demonstrated their potential as sources of bioactive compounds, antioxidant capacity, prebiotic oligosaccharides, and essential amino acids [8,14]. Additional evidence supports the nutritional potential of *Wolffia* sp. as alternative protein sources by Hu et al. [15], who reported that a high-protein strain of *Wolffia* sp. contained 50.89% protein, with a favorable fatty acid profile dominated by unsaturated fatty acids, including palmitic, linoleic, and α-linolenic acids. Similarly, Muslykhah et al. [16] demonstrated that *W. globosa* outperformed several alternative protein sources in terms of phenolic and flavonoid contents, antioxidant activity, and nutrient bioaccessibility, largely due to its low antinutritional factor content as revealed through in vitro digestion studies.

The finding of the current research indicated that riceberry rice yogurt has significant potential for antidiabetic activity to inhibit α-amylase and α-glucosidase activities obtained from their phytonutrients and biological compounds. In accordance with Anuyahong et al. [5], who reported a significant reduction in plasma glucose concentration 30 min after consumption, accompanied by increased ferric reducing antioxidant power (FRAP), Trolox equivalent antioxidant capacity (TEAC), and oxygen radical absorbance capacity (ORAC). Moreover, Ni et al. [17] demonstrated that reformulating yogurt with blackcurrant pomace and salal berry extracts enhanced α-amylase, α-glucosidase, and DPP-IV inhibitory activities during refrigerated storage, which was attributed to both bioactive peptides and sustained lactic acid bacteria viability. Also, their peptidomic analysis identified 486 peptides, including several with known bioactivities such as ACE inhibition and antimicrobial effects. In accordance with Phupaboon et al. [18], who conducted a study on a nutritionally enriched plant-based yogurt formulated with *W. globosa*, the research highlights its potential as a functional food in response to the growing demand for plant-based alternatives. Other reason of these findings is consistent with previous reports the among of formulations tested, WSbY-F4 (15% *W. globosa* supplementation) exhibited the most favorable physicochemical, microbiological, and functional properties (e.g., soluble-protein hydrolysates and soluble peptides), as well as biological capacities in terms of antioxidants: TPC, TFC, DPPH, ABTS, FRAP, and antimicrobial activity (e.g., against *Escherichia coli*), suggesting that the incorporation of *W. globosa* at higher concentrations plays a critical role in enhancing product quality.

In addition, this research aims to prove the degradation process caused by microbial activity, as well as the formation of various metabolites that occur during the fermentation process of plant-based yogurt. The presence of these compounds is consistent with previous studies reporting diverse bioactive metabolites in rice-based and pigmented rice varieties. For example, targeted LC–MS/MS analyses of black rice have identified γ-oryzanol, ferulic acid, 4-hydroxybenzoic acid, apigenin, tricin, avenasterol, coumarin, caffeic acid, α-tocopherol, protocatechuic acid, and dehydroxymyricetin [19]. Similarly, anthocyanins such as cyanidin-3-glucoside and peonidin-3-glucoside have been reported in Thai riceberry rice using GC–MS and LC–ESI–MS/MS analyses [20].

Previous studies have demonstrated that anthocyanin-rich riceberry rice extracts can inhibit intestinal α-glucosidase, particularly maltase and sucrase, and that key anthocyanins such as cyanidin-3-glucoside and peonidin-3-glucoside effectively inhibit pancreatic α-amylase and α-glucosidase [21,22]. While the present study identified indoramin, glimepiride, and vignatic acid B as compounds with strong predicted binding affinities, these findings should be interpreted cautiously, as in silico docking reflects binding potential rather than direct biological activity. Nevertheless, these results provide complementary mechanistic insight into the observed antidiabetic effects of RBRMY-WGP hydrolysates.

Although acarbose is widely used as a clinical α-glucosidase inhibitor, its administration has been associated with gastrointestinal and cardiovascular side effects [23], highlighting the need for alternative, food-derived inhibitors. Several traditional Thai medicinal plants, including *Cinnamomum verum*, *Tinospora crispa*, *Stephania suberosa*, *Andrographis paniculata*, and *Thunbergia laurifolia*, have demonstrated α-glucosidase inhibitory activity and blood glucose–lowering effects. Computational docking studies have identified compounds such as chlorogenic acid, β-sitosterol, ergosterol peroxide, borapetoside A, borapetoside D, stephasubimine, and stephasubine as potential inhibitors [24]. Additionally, Khammuang et al. [25] reported that peptides isolated from Thai red glutinous rice exhibited strong antioxidant activity, with specific peptides showing favorable binding affinities toward DPPH and ABTS radicals in molecular docking simulations. These findings align with prior reporting the released peptides, phytosterols commonly found in *Wolffia* extracts, including campesterol, stigmasterol, β-sitosterol, cycloartenol, and brassicasterol, have been associated with multiple physiological functions, such as anti-inflammatory, antioxidant, antitumor, and antidiabetic activities [9]. Another study has revealed that prunin, as a flavonoid from plant-derived compounds, has antioxidant and anti-inflammatory properties that reduce reactive oxygen species and pro-inflammatory cytokines, thereby limiting tumor progression and influencing the tumor microenvironment [26].

Collectively, these findings support the potential of RBRMY-WGP as a functional fermented food with enriched phytochemical composition and antidiabetic relevance. Additionally, these findings are also consistent with previous reports emphasizing the applicability of SwissADME for evaluating phytochemical-derived small molecules [27]. Among the evaluated ligands, indoramin has been previously characterized as a competitive postsynaptic α1-adrenoceptor antagonist with vasodilatory effects, resulting in reductions in systolic and diastolic blood pressure without consistently inducing reflex tachycardia [28]. Unlike non-selective α-adrenoceptor antagonists such as phentolamine, indoramin does not significantly depress myocardial function when administered intravenously. Its reported local anesthetic and antidysrhythmic properties suggest a membrane-stabilizing effect on myocardial cells, which may contribute to its minimal impact on heart rate [28]. The selectivity of indoramin for α1-receptors allows preserved α2-receptor–mediated inhibition of noradrenaline release, thereby limiting β1-receptor activation in the myocardium and reducing the likelihood of tachycardia [29]. Although the precise mechanisms remain incompletely understood, clinical studies have reported stable hemodynamic profiles in healthy individuals and improved cardiac output and filling pressures in patients with congestive heart failure [29]. Furthermore, glimepiride is a well-established sulfonylurea widely used in the clinical management of type 2 diabetes mellitus. Recent systematic reviews emphasize the importance of comprehensive evaluation of its pharmacokinetics, pharmacodynamics, and drug–drug interactions across different populations to account for variability in therapeutic responses [30]. The favorable in silico ADME properties predicted in the present study are consistent with its known oral bioavailability and clinical efficacy. In contrast, limited pharmacokinetic information is available for vignatic acid B, particularly from in silico studies. This lack of data may reflect the broader challenge associated with characterizing novel or less-studied phytochemicals. Comparable compounds, such as α-lipoic acid (thioctic acid), have been clinically used since the 1950s for the management of diabetic peripheral neuropathy due to their antioxidant properties. However, despite demonstrated efficacy in symptom relief, disease-modifying treatments for diabetic complications remain limited [31]. Further experimental validation is required to clarify the pharmacokinetic behavior and therapeutic relevance of vignatic acid B.

Recent literature highlights ongoing conceptual challenges in defining medicinal foods, particularly the overlapping terms “nutraceuticals” and “functional foods,” first introduced by DeFelice in 1989 [32]. Despite variations among organizations such as The Food Information Council (IFIC), The International Life Sciences Institute of North America (ILSI), and Health Canada, a consistent theme emerges: functional foods are those that provide physiological or health benefits beyond basic nutritional value. Also, these benefits may include disease risk reduction or performance enhancement, often achieved through the inclusion or fortification of bioactive components [33]. Ultimately, emerging insights in medicine have confirmed that dietary supplements, long utilized by health-conscious individuals, hold significantly greater potential for the prevention or even cure of chronic diseases than the extensive range of available prescription medications [34].

## 4. Materials and Methods

### 4.1. Materials and Microorganisms

Riceberry rice milk (RBRM), produced from germinated Riceberry rice, and *W. globosa* powder (WGP), used as plant-based protein and phytonutrient sources, were purchased from a local supermarket in Khon Kaen Province, Thailand. Commercial yogurt starter cultures containing *Lactobacillus delbrueckii* subsp. *bulgaricus* and *Streptococcus thermophilus* were obtained from Dairy LB81-Bulgaria Yogurt (CP-Meiji, Thailand). All organic solvents and analytical-grade reagents used in this study were purchased from Sigma-Aldrich.

### 4.2. Development of Riceberry Rice Milk Yogurt Supplemented with W. globosa Powder (RBRMY-WGP)

**Figure 3:**
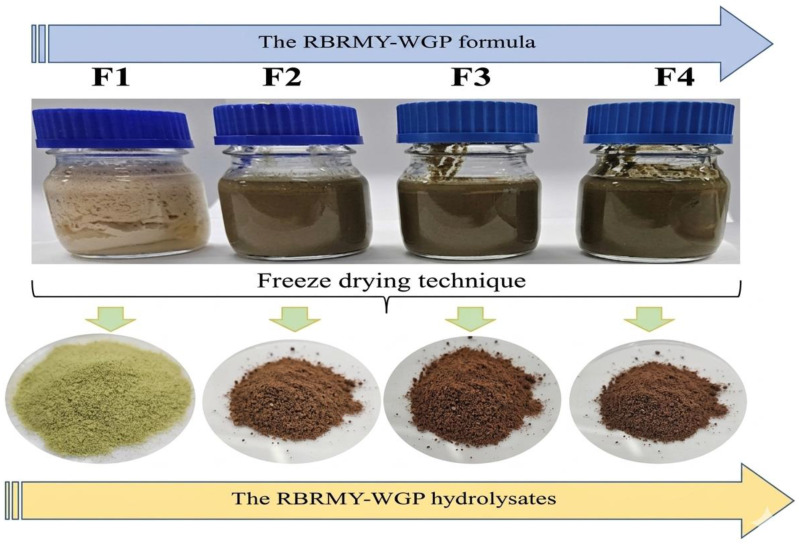
Formulation and hydrolysate products of the different RBRMY-WGP.

Riceberry rice milk yogurt supplemented with WGP (RBRMY-WGP) was prepared by mixing 98% (*v*/*v*) of the pasteurized rice milk supplemented with WGP at different proportions of 0% (F1; control), 5% (F2), 10% (F3), and 15% (F4) as following the protocol of Phupaboon et al. [18]. The mixtures were homogenized and passed through a sieve to ensure uniform dispersion. Approximately 300 mL of each RBRMY-WGP formulation was pasteurized at 80 °C for 12 min, then cooled to 45 °C prior to fermentation. After that, the samples were inoculated with 2% (*w*/*v*) commercial yogurt starter culture (LB81-Bulgaria Yogurt). Fermentation was carried out at 45 °C for 8 h. After fermentation, the yogurts were cooled to room temperature and stored at 4 °C until further analysis. For metabolomics and bioactivity assays, the whole yogurt samples were subsequently homogenized and lyophilized to obtain RBRMY-WGP hydrolysates, which are kept at −20 °C overnight for the freezing step and kept at 45 °C overnight for the drying step (Figure 3).

### 4.3. Determination of Nutritional Composition

The proximate analysis of RBRMY-WGP hydrolysates was conducted in accordance with AOAC procedures [35]. Crude fat content was measured by Soxhlet extraction (Method no. 920.39), crude protein content was determined using the Kjeldahl method (Method no. 2001.11), and carbohydrate-free nitrogen content was calculated by differences (Method no. 986.25). Crude fiber content was quantified according to the AOAC methods no. 991.42 and 993.19. All results were expressed on a dry weight basis (%, *w*/*w*). All measurements were performed in triplicate.

### 4.4. Determination of Phytonutrient Compositions

The phytonutrient composition of lyophilized RBRMY-WGP samples was determined following the methods described by [36]. Total phenolic content (TPC), expressed as mg gallic acid equivalents (GAE)/g of dry sample, was quantified using the Folin–Ciocalteu reagent with absorbance measured at 765 nm. Total flavonoid content (TFC), expressed as mg quercetin equivalents (QUE)/g of dry sample, was determined using a 10% aluminum chloride solution with absorbance measured at 415 nm. Antioxidant activity was evaluated using the DPPH radical scavenging assay, measured at 517 nm, and the ABTS radical cation decolorization assay, measured at 734 nm. Antioxidant capacity was expressed as percentage inhibition. All measurements were performed in triplicate.

### 4.5. Determination of Antidiabetic Activities

The inhibitory activity of α-amylase was evaluated according to the method described by [37], with slight modifications. Seventy μL of p-nitrophenyl-α-D-maltoside, 12 μL of phosphate buffer, and 12 μL of sodium chloride were added to a 96-well microtiter plate. RBRMY-WGP hydrolysates were added to each well to achieve a final concentration of 10 mg/mL. Subsequently, 50 μL of α-Amylase solution (200 U/mL; Sigma-Aldrich, St. Louis, MO, USA) was added, and the absorbance was recorded at 405 nm (A0 min). The mixture was incubated at 37 °C for 180 min, after which the absorbance was measured at 405 nm (A180 min). The inhibition rate of the α-amylase activity was calculated as follows:

(1) $$Inhibition\;rate\left(\%\right)=1-\frac{\left(A\;sample\;180\;min-A\;sample\;0\;min\right)}{\left(A\;blank\;180\;min-A\;blank\;0\;min\right)}\times 100$$

Additionally, the α-glucosidase inhibitory activity was evaluated according to the method described by [38], with minor adjustments. Sample preparation followed the same procedure as that used for the α-amylase inhibition assay. Briefly, 40 μL of α-glucosidase solution (0.1 U/mL; Sigma-Aldrich, St. Louis, MO, USA) and 110 μL of 4-nitrophenyl α-d-glucopyranoside were added to a 96-well microtiter plate. RBRMY-WGP hydrolysates were then added to each well to obtain a final concentration of 10 mg/mL, and the mixture was gently mixed. The absorbance was measured at 405 nm prior to incubation (A0 min). The reaction mixture was then incubated at 37 °C for 15 min, after which the absorbance was measured again at 405 nm (A15 min). The inhibition rate of the α-glucosidase activity was calculated as follows:

(2) $$Inhibition\;rate\left(\%\right)=1-\frac{\left(A\;sample\;15\;min-A\;sample\;0\;min\right)}{\left(A\;blank\;15\;min-A\;blank\;0\;min\right)}\times 100$$

The IC_50_ values of acarbose from porcine pancreas were assessed on α-amylase and α-glucosidase derived from *S. cerevisiae* at final concentrations of 5, 10, 20, 40, and 50 μg/mL. The IC_50_ is the concentration necessary for an inhibitor to decrease enzyme activity by 50%. The IC_50_ value was derived from a dose-dependent activity versus concentration graph, in which data points for both standards at five distinct concentrations were fitted into a non-linear sigmoid curve, reflecting the non-linear concentration dependence of enzyme-inhibitor interactions at both low and high concentrations [38].

### 4.6. Metabolomics Analysis of RBMY-WGP Hydrolysates

Widely targeted metabolomics analysis was performed to compare the metabolite profiles of RBRMY-WGP hydrolysates from the formulation with the highest bioactivity in (F4) and the control formulation (F1). Analysis was carried out using UPLC-QTOF (Waters Xevo G2-XS QTOF MS). Firstly, the lyophilized sample was dissolved in LC/MS solvents and cooled acetonitrile to obtain a final concentration of 20 mg/mL. Then, the supernatant was filtered through a 0.22 μm hydrophilic PTFE syringe filter. An aliquot of 1.0 mL was transferred into an HPLC vial for UPLC-QTOF MS analysis, following the procedure described by [39]. Subsequently, the UPLC-QTOF analysis was performed using a Waters Xevo G2-XS QTOF MS system [40]. The analytical condition was as follows: (I) chromatographic separation was achieved using a CORTECS C_18_ column (2.1 mm × 100 mm, 1.6 µm) coupled with a CORTECS T3 VanGuard column (2.1 mm × 5 mm, 1.6 μm, Waters Pacific Pte. Ltd., Drinagh, Ireland); (II) electrospray ionization (ESI) was operated in both positive and negative ion modes, with capillary voltages set at 2.5 kV and 2.3 kV, respectively; (III) the injection volume for each sample was 3 µL, and the total run time was 20.1 min; and (IV) gradient elution was performed using mobile phase A (0.1% formic acid in water) and mobile phase B (acetonitrile) at a flow rate of 0.4 mL/min at 30 °C.

### 4.7. Computational and In Silico Studies

In silico analysis was performed using a molecular docking approach following the protocol described by [27]. This analysis evaluated the inhibitory potential of 88 selected compounds identified from the RBRMY-WGP hydrolysates (F4) against α-amylase and α-glucosidase, key enzymes associated with type 2 diabetes mellitus (T2DM). These ligand molecules were represented by distinct SMILES structures, which were retrieved based on PubChem ID. Protein targets used in this study included α-amylase from *A. niger* (PDB ID: 2GVY) and α-glucosidase from *S. cerevisiae* (PDB ID: 3A4A), obtained from the Protein Data Bank. Molecular docking simulations were conducted to assess ligand binding affinities at the active site of the target proteins using the SwissDock web server (https://www.swissdock.ch/) accessed on 22 December 2025 via the AutoDock (SwissDock v2024) Vina platform [41]. For further in silico evaluation, the physicochemical and pharmacokinetic properties of compounds exhibiting high binding affinities were predicted using the SwissADME (SwissDock v2024) web tool (http://www.swissadme.ch/) accessed on 22 December 2025. Predictions focused on molecular weight (MW), lipophilicity (Consensus Log Po/w), water solubility (Log S), gastrointestinal absorption, blood–brain barrier (BBB) permeation, and drug-likeness based on Lipinski’s rule of five [27,36].

### 4.8. Statistical Analysis

All experiments in this study were conducted in triplicate (*n* = 3). Data were presented as mean ± standard deviation (SD). Analysis of variance (ANOVA) was employed to assess differences among treatments, followed by Duncan’s new multiple range test, with statistical significance set at *p* < 0.05. Statistical analyses were conducted using IBM SPSS Statistics Version 27.0. For metabolomics analysis, UPLC-QTOF data were processed using Progenesis QI (v3.0) software in conjunction with the METLIN database to identify untargeted small molecules. The resulting datasets were subsequently exported to EZinfo (v3.1) program for multivariate statistical analysis.

## 5. Conclusions

The present study investigated the effects of supplementing RBRMY with varying proportions of WGP as a source of bioactive compounds with antioxidant and antidiabetic properties. Among the formulations, the plant-based yogurt containing 15% WGP (F4) exhibited the most favorable nutritional profile, characterized by high dietary fiber and protein contents and a relatively low-fat content. In addition, F4 demonstrated enhanced functional properties, including elevated total phenolic and flavonoid contents, strong DPPH and ABTS radical scavenging activities, and pronounced inhibitory effects against α-amylase and α-glucosidase. Metabolomics analysis combined with molecular docking revealed strong predicted binding affinities of selected phytochemical compounds, including indoramin, glimepiride, and vignatic acid B, toward the active sites of α-amylase and α-glucosidase, suggesting potential mechanistic contributions to the observed antidiabetic activity. These interactions were primarily mediated through hydrogen bonding and hydrophobic interactions, which are important for enzyme inhibition. Furthermore, in silico ADME evaluation indicated that these compounds possess physicochemical and pharmacokinetic properties consistent with acceptable drug-likeness criteria, supporting their potential applicability in functional food development. Overall, this study provides novel nutriomic insights into the development of high-value plant-based yogurt enriched with *W. globosa*. The findings highlight the potential of RBRMY-WGP as a functional fermented food with enhanced nutritional quality and bioactive properties. Nevertheless, further in vivo and clinical studies are required to validate the bioavailability, safety, and health benefits of these compounds before practical application in dietary interventions or food supplementation.

## Figures and Tables

**Table 1:** Bioactive compounds and antidiabetic activities of RBMY-WGP hydrolysates.

Assess	RBRMY-WGP Formula ^1^			
	F1	F2	F3	F4
**Bioactive compounds**				
Total phenolic content (mg GAE/g)	20.09 ± 0.35 ^d^	22.31 ± 0.13 ^c^	28.33 ± 0.87 ^b^	33.70 ± 0.13 ^a^
Total flavonoid content (mg QUE/g)	11.13 ± 0.12 ^d^	13.87 ± 0.23 ^c^	20.13 ± 0.01 ^b^	25.20 ± 0.35 ^a^
DPPH inhibition (%)	33.03 ± 0.52 ^d^	39.55 ± 0.79 ^c^	40.30 ± 0.26 ^b^	41.52 ± 0.95 ^a^
ABTS inhibition (%)	12.12 ± 0.97 ^d^	15.76 ± 0.93 ^c^	31.97 ± 0.17 ^b^	78.18 ± 0.10 ^a^
**Antidiabetic activities**				
α–amylase inhibition (IC_50_, mg/mL)	2.42 ± 0.08 ^d^	1.24 ± 0.06 ^c^	0.95 ± 0.03 ^b^	0.89 ± 0.05 ^a^
α–glucosidase inhibition (IC_50_, mg/mL)	1.56 ± 0.25 ^c^	1.47 ± 0.44 ^b^	1.46 ± 0.36 ^b^	1.32 ± 1.03 ^a^

^1^ Data are expressed as mean ± SD (*n* = 3). Different letters (a–d) within the same column indicate statistically significant differences (*p* < 0.05).

**Table 2:** Phytochemical compounds identified by UPLC-QTOF in RBMY-WGP hydrolysates and their molecular binding interactions with α-amylase (PDB ID: 2GVY, *A. niger*) and α-glucosidase (PDB ID: 3A4A, *S. cerevisiae*).

No.	Mode	RT (min)	*m*/*z*	Identified Compounds	Formula	PubChem ID	Calculated Affinity (kcal/moL)	
							2GVY	3A4A
1	+	1.728	513.1225	Leucodelphinidin 3-O-α-L-rhamnopyranoside	C_21_H_24_O_12_	44257158	−7.266	−6.779
2	+	17.880	469.1392	Macaflavone II	C_26_H_26_O_6_	44258686	−8.940	−6.793
3	+	17.126	644.2431	Acarbose	C_25_H_43_NO_18_	9811704	−7.244	−6.042
4	+	17.735	518.2916	Vignatic acid B	C_27_H_41_N_3_O_7_	85261327	−5.396	−8.350
5	+	18.169	504.2423	Cerivastatin	C_26_H_34_FNO_5_	446156	−6.871	−1.126
6	+	19.422	229.0876	Dihydroresveratrol	C_14_H_14_O_3_	185914	−7.415	−7.065
7	+	20.1	293.1797	Embelin	C_17_H_26_O_4_	3218	−7.107	−6.428
8	+	18.065	535.2239	Glimepiride	C_24_H_34_N_4_O_5_S	483928029	−9.867	−1.914
9	−	10.179	316.1918	Nateglinide	C_19_H_27_NO_3_	5311309	−7.767	−6.427
10	−	18.049	341.1717	Acitretin	C_21_H_26_O_3_	5284513	−7.244	−5.652
11	−	19.273	507.2044	Mangostenone B	C_28_H_30_O_6_	21672078	−5.487	−6.819
12	−	20.802	392.2023	Indoramin	C_22_H_25_N_3_O	33625	−9.155	−8.216

**Table 3:** Physicochemical properties and ADME profiles of the selected potential antidiabetic compounds identified in RBRMY-WGP hydrolysates.

Characteristics	Ligands/Compounds		
	Indoramin	Glimepiride	Vignatic Acid B
**Physicochemical properties**			
Molecular weight (g/moL)	347.45	490.62	519.63
Num. H-bond acceptors	2	5	7
Num. H-bond donors	2	3	5
Num. rotatable bonds	6	11	9
**Lipophilicity**			
Consensus Log P_o/w_	3.43	2.84	2.09
**Water Solubility**			
Log S (ESOL)	−4.46	−4.71	−4.98
Solubility class	Soluble	Soluble	Soluble
**Pharmacokinetics**			
GI absorption	High	Low	Low
BBB permeant	Yes	No	No
P-gp substrate	Yes	Yes	Yes
**Drug-likeness**			
Lipinski violation	Accepted	Accepted	Accepted
**Medicinal Chemistry**			
Synthetic accessibility	Very good	Good	Good

## Data Availability

The original contributions presented in this study are included in the article/Supplementary Material. Further inquiries can be directed to the corresponding author.

## Supplementary Materials

The following supporting information can be downloaded at: https://www.mdpi.com/article/10.3390/ijms27104256/s1.

## Abbreviations

The following abbreviations are used in this manuscript:

RBRM	Riceberry rice milk
WGP	*Wolffia globosa* powder
RBRMY-WGP	Riceberry rice milk yogurt supplemented with *Wolffia globosa* powder
T2D	Type 2 diabetics
DPPH assay	2,2-diphenyl-1-picrylhydrazyl
ABTS assay	2,2-azino-bis-3-ethylbenzothiazoline-6-sulphonic acid
FRAP assay	Ferric reducing antioxidant power
TPC	Total phenolic content
TFC	Total flavonoid content
GAE	Gallic equivalence
QUE	Qurcetin equivalence
IC_50_	Inhibitory concentration of 50%
LC-MS/MS	Liquid chromatograph mass spectrometer
LC-QTOF	Liquid chromatograph-quadrupole time of flight
UPLC-QTOF	Ultra performance liquid chromatograph-quadrupole time of flight
LC-ESI-MS/MS	Liquid chromatography- electrospray ionization- mass spectrometry
GC-MS/MS	Gas chromatograph mass spectrometer
mg GAE/g DW	Milligrams of gallic acid equivalent per gram of dry weight
mg QUE/g DW	Milligrams of quercetin equivalent per gram of dry weight
% DW,	Percentage of dry weight
