# Supplementary material for: Characterization of Metabolites in Plant-Based Milk Yogurt Enriched with Wolffia globosa to Improve Bionutritional and Functional Properties
Source: Int J Mol Sci. 2026 May 10;27(10):4256. doi: 10.3390/ijms27104256 (PMC13206772; doi:10.3390/ijms27104256)
Supplement: Supplementary file 1 [file ijms-27-04256-s001.zip › ijms-4268930-supplementary.pdf]

Supplementary Materials

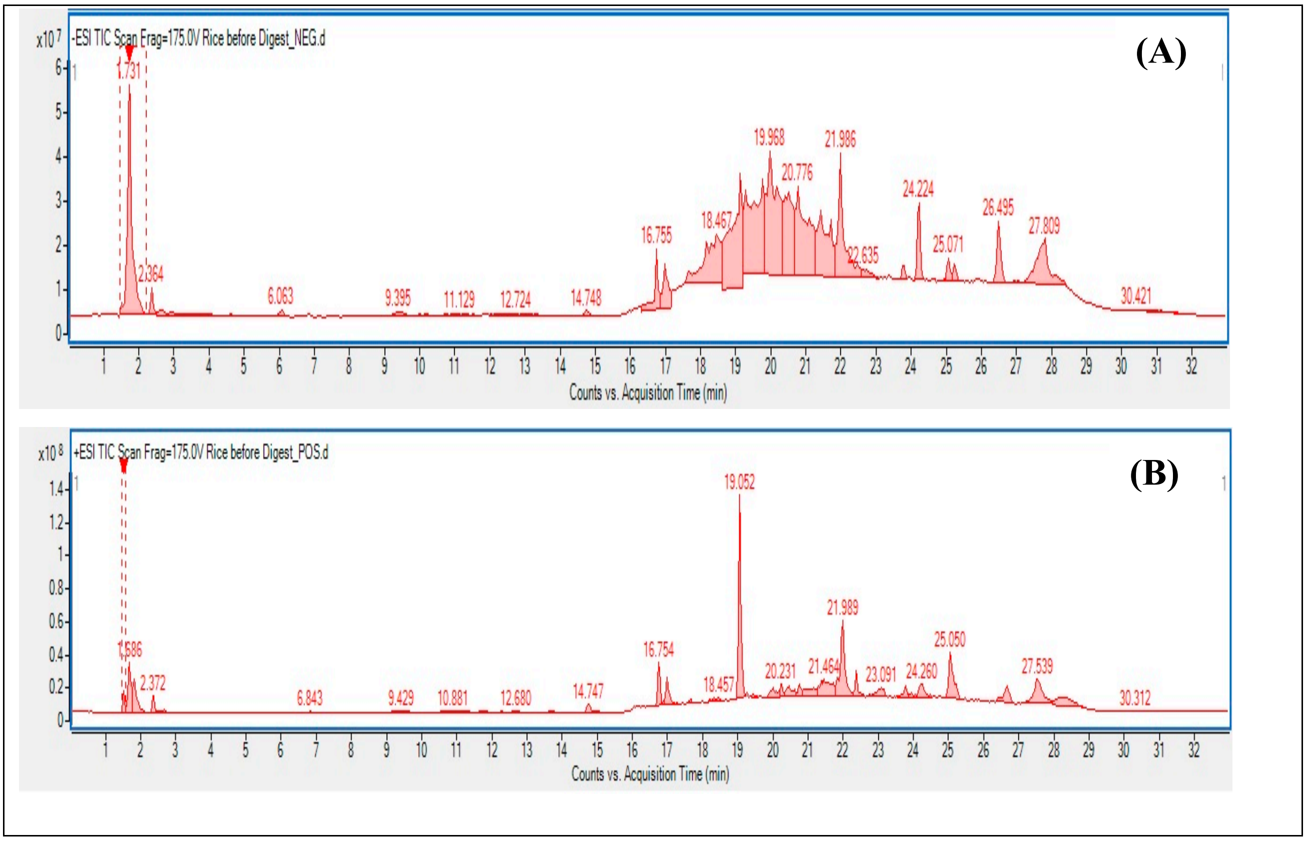

Figure S1. UPLC–QTOF based peak chromatograms of RBMY-WGP hydrolysates (F1, control) acquired in (A) ESI negative mode, and (B) ESI positive mode.

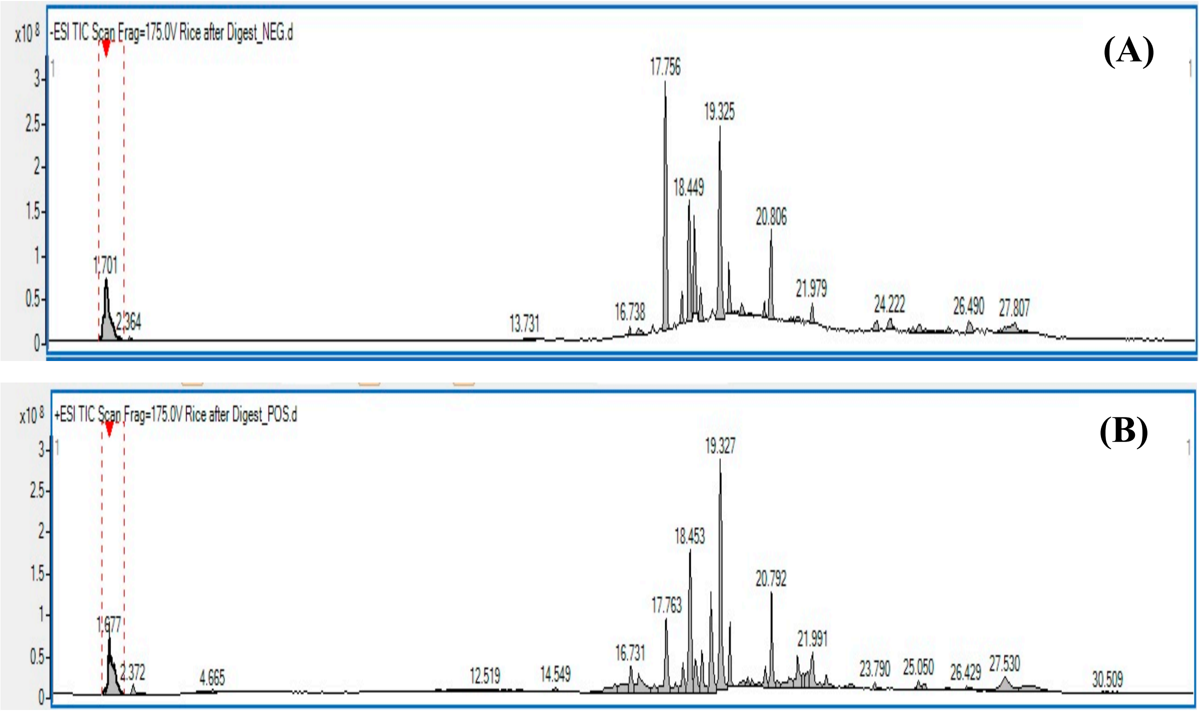

Figure S2. UPLC–QTOF based peak chromatograms of RBMY-WGP hydrolysates (F4, riceberry milk yogurt supplemented with 15% *Wolffia globosa*) acquired in (A) ESI negative mode, and (B) ESI positive mode.

(a-1)

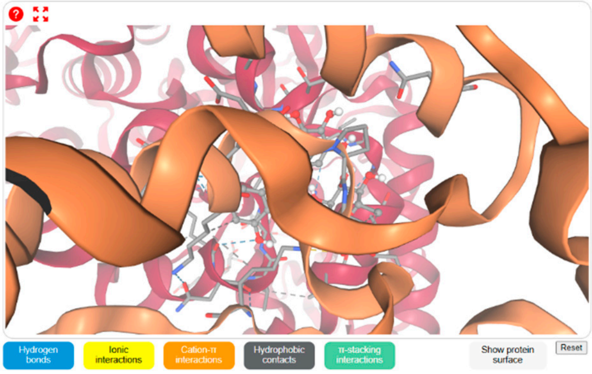

(a-2)

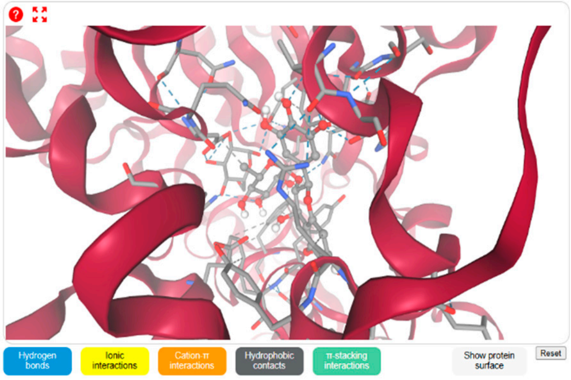

(b-1)

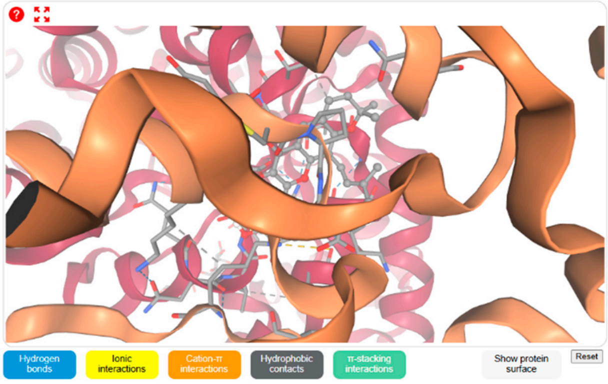

(b-2)

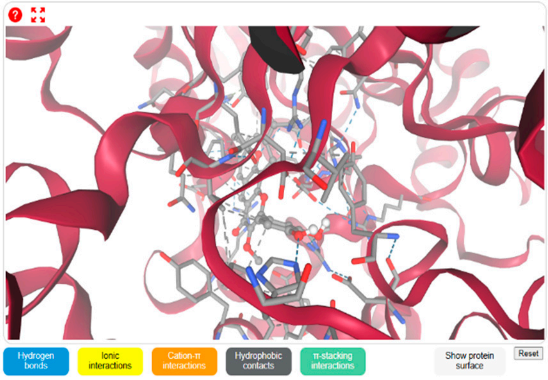

(c-1)

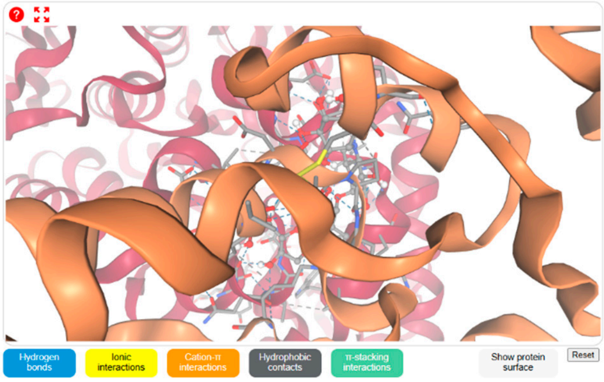

(c-2)

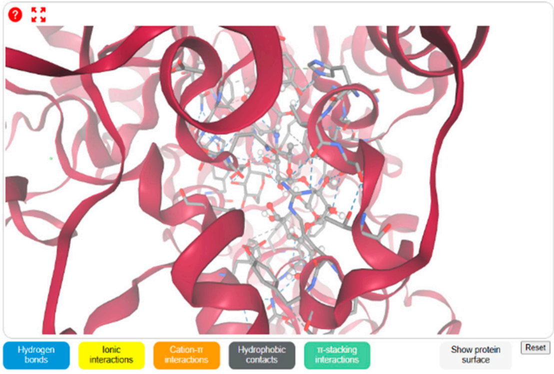

(d-1)

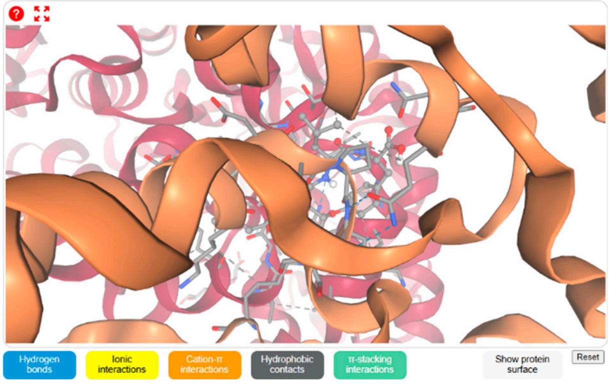

(d-2)

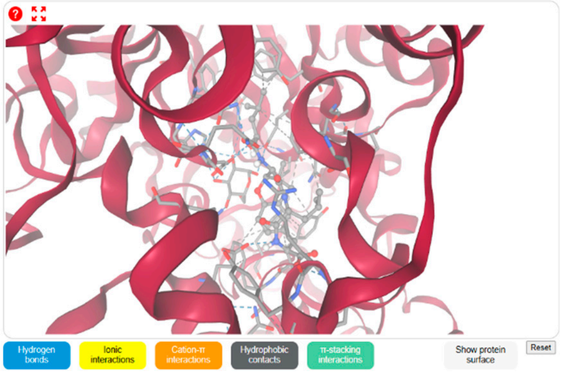

(e-1)

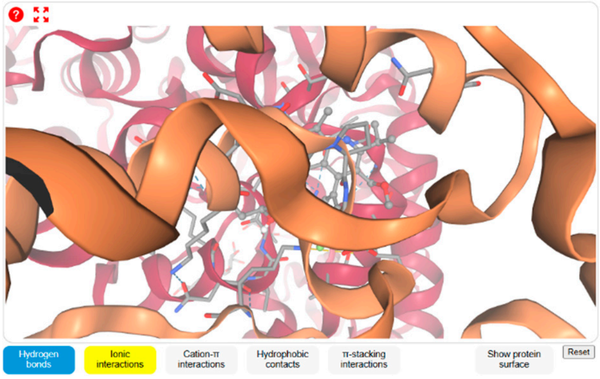

(e-2)

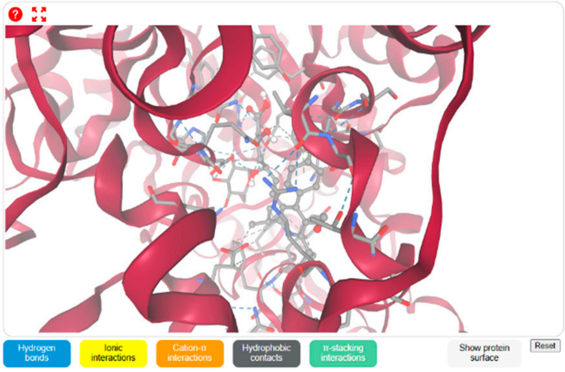

(f-1)

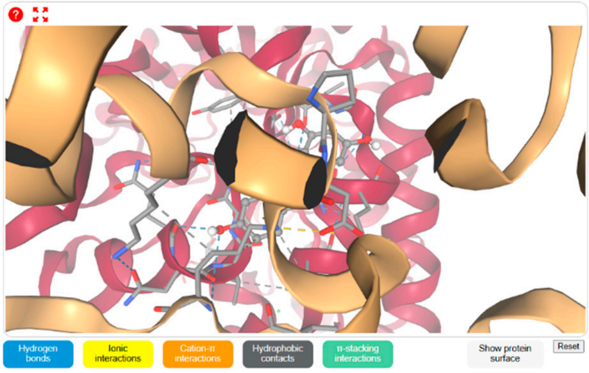

(f-2)

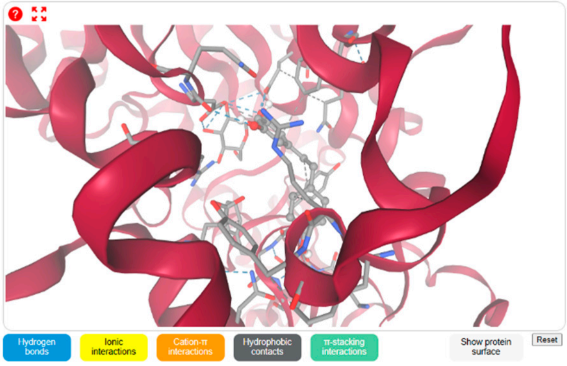

(g-1)

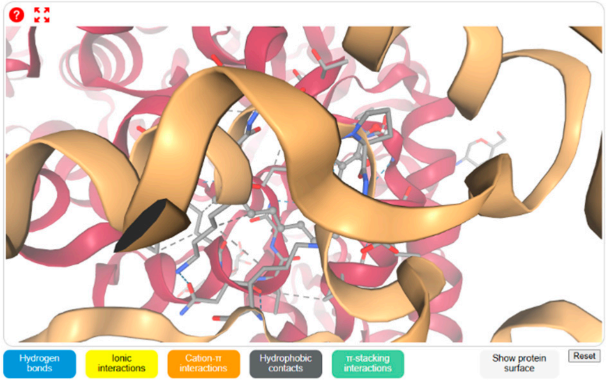

(g-2)

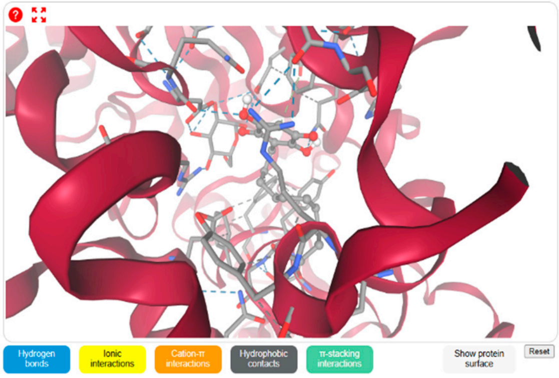

(h-1)

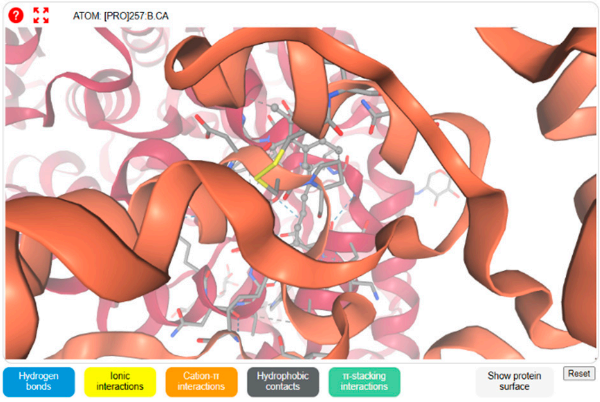

(h-2)

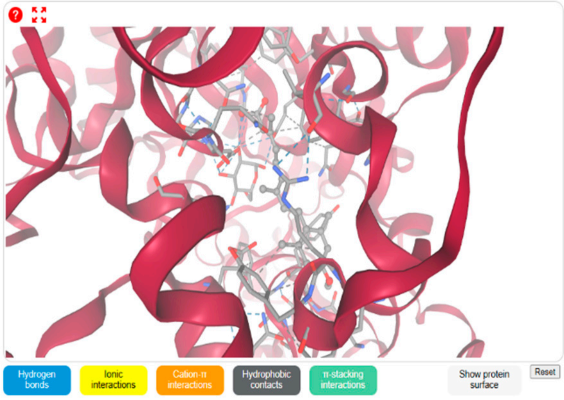

(i-1)

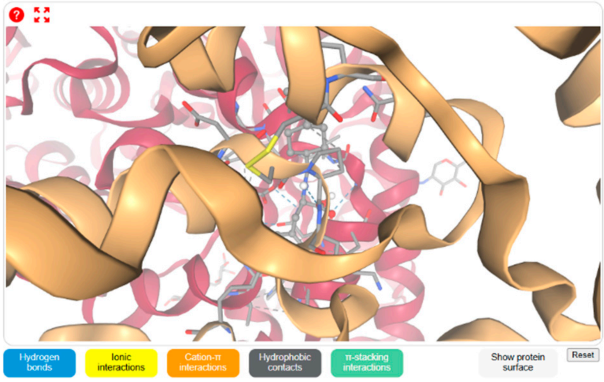

(i-2)

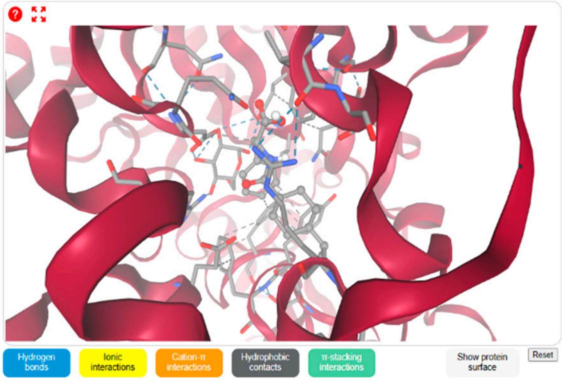

(j-1)

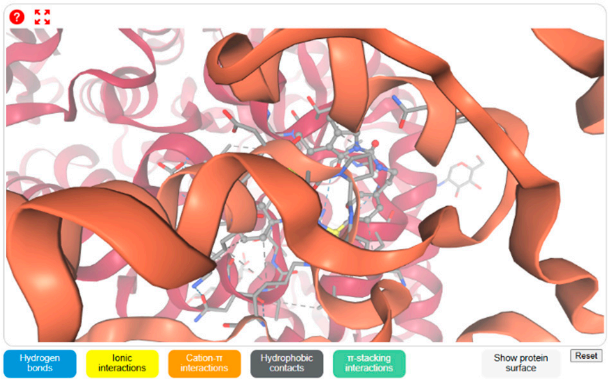

(j-2)

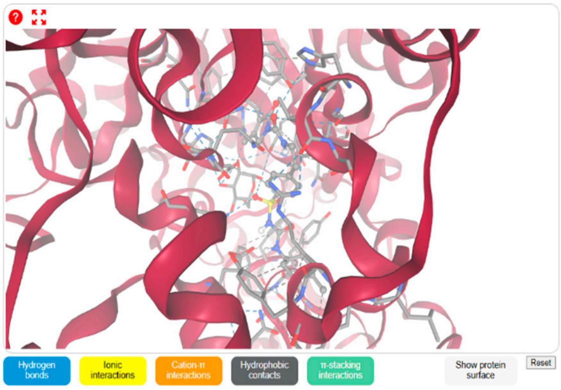

(k-1)

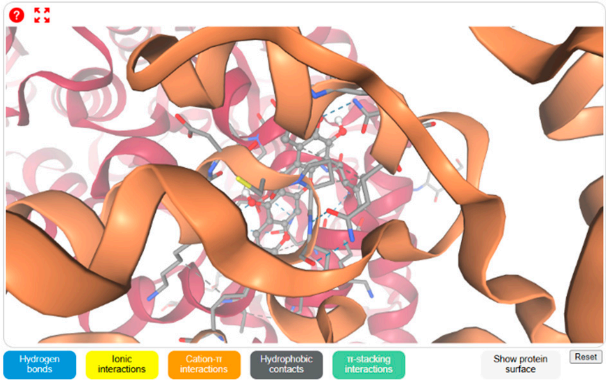

(k-2)

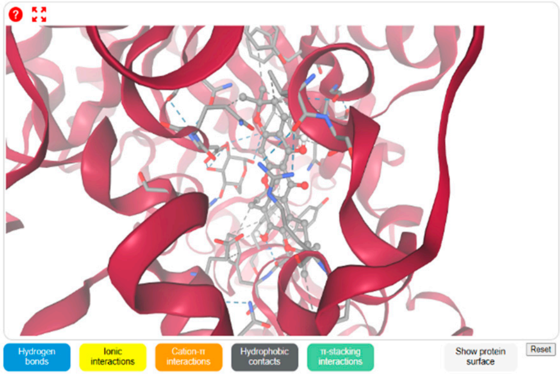

(l-1)

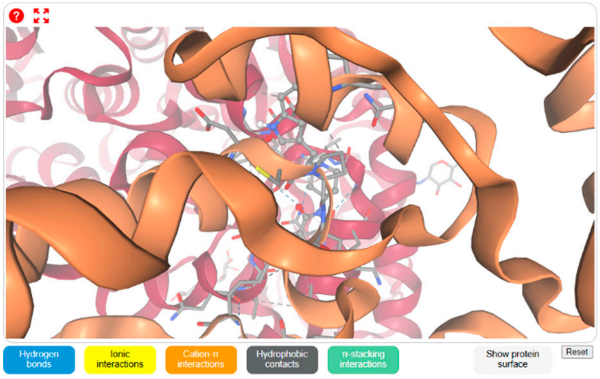

(l-2)

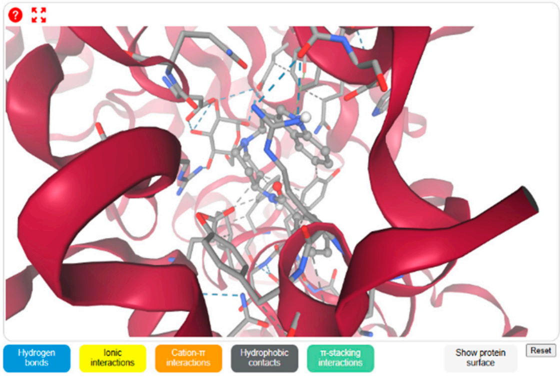

Figure S3. Three-dimensional molecular docking models and surface representations of protein-ligand binding interactions for the selected ligands (ligands 1–12, as listed in Table 2). Docking interactions

with α-amylase (PDB ID: 2GVY) are shown in panels a-1 to l-1, and interactions with α-glucosidase (PDB ID: 3A4A) are shown in panels a-2 to l-2.

Table S1. Eighty-eight phytochemical compounds identified by UPLC–QTOF after filtering 1,188 features detected in negative ion mode, and 989 features detected in positive ion mode, and their associated pharmacological and biological activities.

No.	Compounds	Mode	RT	*m/z*	Effects
1	Leucodelphinidin 3-O-α-L-rhamnopyranoside	+	1.728	513.1225	Lowering blood sugar
2	Gossypetin 8-glucoside-3-sulfate	+	1.77	559.0432	Anti-oxidant and improves cardiovascular function
3	Chondroitin 6-sulfate	+	1.966	917.1179	Chondroitin sulphate in patients with osteoarthritis is possibly the result of the stimulation of the synthesis of proteoglycans and the decrease in catabolic activity of chondrocytes by inhibiting the synthesis of proteolytic enzymes and other factors that contribute to cartilage matrix damage and cause the death of these cells.
4	Taurine	+	19.689	482.2958	Taurine has role in retinal health and anti-inflammatory
5	Piperacillin β-lactam	+			A beta-lactam/beta-lactamase inhibitor
6	Crizotinib	+	1.76	494.1197	Attenuates lung cancer metastasis
7	Duloxetine	+	1.978	342.1137	Treatment for major depressive disorder
8	Lappaol A	+	9.894	581.2037	Anti-cancer, neuroprotection, anti-rheumatic, and aphrodisiac
9	Isoneotheaflavin	+	17.061	563.1198	Useful to remove toxic amyloid deposits in Alzheimer's and Parkinson's disease brains.
10	Macaflavone II	+	17.880	469.1392	Strong antibacterial and also antioxidant activities. More importantly, the *in vivo* assay exhibited the ability of 1 to decrease blood glucose levels in the diabetic rats to the normal level.
12	Ophiopogonin C	+	16.73	853.4612	-Significantly ameliorates radiation-induced pulmonary fibrosis and may be a promising therapeutic strategy for this disorder.<br>-Owners the amount of collagen in lung tissue, a key component of fibrosis.

					-It increases the activity of antioxidant enzymes like SOD (superoxide dismutase) and decreases oxidative stress markers like MDA (malondialdehyde) in the lungs.
13	Licoricesaponin G2	+	16.492	837.392	Suppressing TNF-α signalling pathway activation pulmonary fibrosis
14	Acarbose	+	17.126	644.2431	α-glucosidase inhibitor that is commonly used to control postprandial blood glucose.
15	Ergotamine	+	17.421	580.2566	Treatment of migraine
16	Azelaic acid	+	17.178	187.0976	Azelaic acid (AzA), a saturated dicarboxylic acid, is indicated for the treatment of acne vulgaris, rosacea, melasma, and post-inflammatory hyperpigmentation. Its antimicrobial, anti-inflammatory, and antimelanogenic properties support its use;
17	Tauroursocholic acid	+	17.011	514.2845	Neuroprotective agent
18	Glycocholic acid	+	18.114	464.3025	Anti-inflammatory and anti-bacterial
19	Prostaglandin E2 p-acetamidophenyl ester	+	17.287	530.2794	Anti-inflammatory
20	Vignatic acid B	+	17.735	518.2916	Anti-diabetes
21	Glimepiride	+	18.065	535.2239	Reduces blood glucose levels and increases insulin levels in blood.
22	Cerivastatin	+	18.169	504.2423	LDL cholesterol decreases, total cholesterol decreases and triglyceride decreases
23	Lucidenic acid B	+	18.803	473.2522	Induces apoptosis in human leukemia
24	Ganolucidic acid A	+	19.157	545.3156	Anti-inflammatory and anti-tumor activities
25	Dihydroresveratrol	+	19.422	229.0876	Treatment of obesity and type 2 diabetes and anti-oxidative suppress
26	Chrysophanol 8-(6-galloylglucoside)	+	19.351	567.113	Antiviral activity and subsequently their potential to serve as drug candidates for further designing of target-specific anti-COVID-19 agents
27	Isobutyl salicylate	+	19.479	193.0872	Used in cosmetics
28	Embelin	+	20.1	293.1797	-Anti-inflammatory and anti-tumor effects in various cancer cells, activating tumor suppressor gene p53 and inducing apoptosis

					-Significantly improve the recognition index and memory retention in treat AD<br>-Antidiabetic activity
29	Myrsinone	+	19.841	293.1762	Free-<br>radical scavenging, melanogenes is inhibition, and pollution defines properties, positioning it as a promising ingredient in skincare
30	Glutathione Cobalamin	+	1.627	816.8202	Inhibited intracellular peroxide production, maintained intracellular glutathione levels, and prevented apoptotic and necrotic cell death.
31	Histidinyl-Cysteine	+	18.28	303.0771	Powerful neuroprotective agent.
32	Docusate	+	19.311	421.2275	Docusate is effective in the treatment of<br>constipation in pediatric patients.
33	Tibolone	+	22.209	311.2017	Based on the evidence available, tibolone is a valuable treatment option to relieve menopausal complaints, especially in women suffering persistent fatigue, blunted motivation, and loss of sexual desire despite an adequate estrogen replacement.
34	Vitamin D derivatives (6RS / 6R	+	20.919	467.3015	The (6R) and (6S) are mirror images of each other at the C6 position. anti-proliferative and pro-differentiation effects in precursor cell lines and malignant cell types.
35	Armillarivin	+	21.149	383.1849	Exhibit Anti-cancer activity
36	Fasciculic acid A	+	21.518	619.4209	Potent acetylcholinesterase inhibitors
37	Myricetin 3,7-diglucuronide	+	24.139	669.0845	Downregulated the UVA-induced pro-inflammatory cytokines in keratinocytes
38	Lisuride	+	26.521	337.2057	Improve treatment of central dopaminergic in Parkinson's disease
39	Tabernamine	+	21.811	661.3841	Anti-cancer activity
40	Propranolol	+	22.208	258.1502	A primary choice for essential tremor treatment.
41	Methacycline	+	27.551	441.1273	Anti-bacterial activity
42	Leucomycin A8	+	20.927	391.7104	Anti- viral (anti-influenza A virus)
43	Fosinopril	+	18.425	578.2932	Exhibits antiamnesic activity, indicating its possible role in preventing memory deficits
44	BAY-60-7550	+	19.964	521.2439	-Aβ-oligomers (Aβo)<br>-Induced neuronal damage and memory impairment

					-Improves neuronal remodelling and decreases oxidative damage in Aβ-treated mice.
45	Resveratrol 4'-(2-galloylglucoside)	-	1.881	541.1319	Anticancer effect against leukaemia cells via inducing apoptosis
46	Epicatechin 3-O-(4-methylgallate)	-	1.798	455.1013	Has free radicals scavenging effects
47	Hesperetin 7-O-glucoside	-	1.911	509.1351	Has strong anti-inflammatory activity *in vitro* and *in vivo* and potential preventive or therapeutic effects for chronic inflammation diseases.
48	Malvidin 3-(6-malonylglucoside) 5-glucoside	-	1.767	741.1829	The inhibition of hepatic inflammation and oxidative stress and the modulation of the abundance of probiotic microbial genera
49	Nateglinide	-	10.179	316.1918	Control diabetes based on the effect on hyperglycemia
50	Cabergoline	-	18.162	450.2864	Improvement in the speed of processing, working memory, visual learning and reasoning and problem-solving domains
51	Pergolide sulfone	-	6.272	391.1698	Dopamine agonist activities of pergolide
52	Mycalamide A	-	16.779	548.2696	Antitumor activity
53	Ophiopogonin D	-	16.747	853.4577	Effect on bone protection, cardiovascular protection, immune regulation, anti-cancer, anti-atherosclerosis, and anti-inflammatory
54	Elatoside E	-	16.445	881.4759	Decrease plasma glucose level by oral sugar tolerance test in rats
55	1-O-Sinapoylglucose	-	1.739	385.1179	Serve as an acyl donor in acylation of anthocyanins and generate cyanidin 3-xylosyl (sinapoylglucosyl) galactoside in purple carrots
56	3-Aminopropanesulphonic acid	-	1.721	138.0248	An inhibitor of the enzyme glutamic acid decarboxylase on prolactin secretion
57	N4-Phosphoagmatine	-	16.985	209.0796	Induced neuroprotection includes anti-oxidation, anti-apoptosis, anti-inflammation, brain blood barrier (BBB) protection and brain edema prevention.
58	Sertraline carbamic acid glucuronide	-	1.7	524.0882	Treat several psychiatric disorders including major depressive disorder, panic,generalized

					and social anxiety disorders as well as obsessive-compulsive disorder (OCD).
59	Crizotinib	-	1.748	494.1205	Anti-tumor and anti-lung cancer
60	Retapamulin	-	17.16	562.3226	Antibacterial approved for the treatment of uncomplicated superficial skin infections.
61	Estazolam	-	17.634	293.0635	Significant improvements in memory, wake time after sleep onset and total sleep time
62	Acitretin	-	18.049	341.1717	Improving insulin resistance and has a glucose reducing effect.
63	Triamcinolone hexacetonide	-	16.623	531.2794	Protective effect against cartilage damage and osteophyte formation
64	Latanoprost ethyl amide (Latanoprost Analog)	-	17.572	462.2853	Significantly reduces intraocular pressure and increases ocular blood flow. These effects may be beneficial in the management of glaucoma patients.
65	Tafluprost ethyl amide (prostaglandin derivative)	-	1.807	482.2343	Potent antiglaucoma agents
66	Capreomycin	-	14.216	667.343	Second-line drug for multiple-drug-resistant tuberculosis (TB)
67	Premithramycin A2'	-	1.701	719.2079	An intermediate in the production of mithramycin. The antitumor antibiotic mithramycin A (MTA) binds to G/C-rich DNA sequences in the presence of dications.
68	Tetracenomycin F2	-	1.747	383.0753	Tetracenomycin F2 is a precursor in the biosynthetic pathway of the antibiotic tetracenomycin C
69	N-Oleoyl Dopamine	-	19.573	462.3204	A novel therapeutic targets for treating ocular diseases involving retinal fibrosis and fibrotic pathologies in other organ systems.<br>2-potential brain modifier of motor behavior, the biological consequences of which remain to be explored.
70	Mangostenone B	-	19.273	507.2044	A neuroprotective, anti-proliferative, antinociceptive, antioxidant, pro-apoptotic, anti-obesity, anti-inflammatory, and hypoglycemic
71	Verapamil	-	19.322	453.2845	A calcium channel blocking drug, terminates supraventricular arrhythmias and treat hypertention
72	Fluphenazine enanthate	-	18.475	594.2614	Treatment of schizophrenia
73	Clarithromycin	-	19.583	746.4655	Antituberculosis activity

74	Indoramin	-	20.802	392.2023	Effective in lowering blood pressure
75	Rifaximin	-	20.804	391.6761	Potential activity in a multitude of gastrointestinal diseases.
76	Sufentanil	-	21.373	431.2021	Induction and maintenance of anaesthesia,
77	Ciclesonide	-	19.447	539.3047	Preventative treatment of persistent bronchial asthma in adults
78	Methazolamide	-	19.111	235.0001	Reduces hypoxic pulmonary vasoconstriction
79	4-Ketolutein F	-	18.224	581.4049	Specific keto-carotenoid, which is a modified version of the pigment lutein, physically quench singlet oxygen and scavenge peroxyl radicals,
80	3,5-Dihydroxyphenyl 1-O-(6-O-galloyl-beta-D-glucopyranoside)	-	1.726	439.0894	Potential antioxidant, anti-inflammatory, and anticancer effects
81	Squamosinin A	-	27.265	621.4368	Contribute to improved physical fitness and enhanced exercise performance.
82	Taraxacolide 1-O-b-D-glucopyranoside	-	24.215	427.1989	Potent antileishmanial agents
83	Ecabet	-	23.823	379.1591	-Prevents esophageal lesions induced by the reflux of gastric juice in rats<br>-Protective effects on the gastric mucosa has anti-Helicobacter pylori effects, binding with urease to inhibit H. pylori activity, and causing the bacterial to become non-viable
84	Quinagolide		26.506	440.2219	Reduced tumour blood supply and reduce tumour blood flow, which may be
85	Ramelteon	-	22.062	258.149	Decrease the duration of ICU stay as well as decreased the occurrence rate and duration of delirium statistically significantly
86	Jurubine	-	25.06	594.3948	Neuroprotection and the prevention and treatment of cardiovascular diseases
87	Brosimacutin C	-	24.196	341.1385	Cytotoxic activity against murine leukemia P388 cells
88	9(S)-HpOTrE	-	21.985	309.2065	Anti-inflammatory effect
